# Supplementary material for: Disposal practices of unused and expired pharmaceuticals among the general public in Quetta city, Pakistan
Source: PLoS One. 2022 May 19;17(5):e0268200. doi: 10.1371/journal.pone.0268200 (PMC9119513; doi:10.1371/journal.pone.0268200)
Supplement: S1 File — (DOCX) [file pone.0268200.s001.docx]

**Disposal practices of unused and expired pharmaceuticals among the general public in Quetta city, Pakistan**

**Section A: Demographic information**

| Age (years) |  | |
| --- | --- | --- |
| Gender | Male | Female |
| Marital status | Married | Unmarried |
| Locality | Urban | Rural |
| Education | Primary | Secondary |
|  | Higher secondary | Graduate |
| Occupation | Unemployed | Housewife |
|  | Public employee | Business |
| Income (Pakistan Rupee) | None | > 20000 |
|  | 20001-40000 | > 40000 |

**Section B: Storage, and disposal practices of unused medicines**

| Do you currently have any unused medicines stored at home? | Yes | No |
| --- | --- | --- |
| Why do you keep unused medicines at home? | Can reuse the medicine | |
|  | Medicine is needed in an emergency | |
|  | Can be used by friends or family members | |
| Do you know how to store medicines at home? | Yes | No |
| Do you read storage instructions on the labels/leaflets? | Yes | No |
| Where do you store your unused medicine? | Kitchen cabinet | Bathroom cabinet |
|  | Bedroom cabinet | Medicine box |
|  | Refrigerator | |
| Do you dispose of unused medicines stored at your home? | Yes | No |
| How do you dispose of unused medicines stored at your home? | The exchange at the  pharmacies | Give to hospitals  /clinic |
|  | Throw away in dustbins (household trash) | Give to friends  or relatives |

**Section C: Practices and disposal of expired medicines**

| Do you check the expiry date of the medicines before purchasing? | Yes | No |
| --- | --- | --- |
| Do you check the expiry date before using the medicines? | Yes | No |
| Do you know the procedure to dispose of nearly expired medicines? | Yes | No |
| How do you dispose of nearly expired medicines? | Donte to hospital  /clinic | Return it to  pharmacies |
|  | Throw away in dustbins (household trash) | Flush in toilet  or sink |
|  | Give them to  friends/family/  members/ others | Keep at home  until expired |
| Do you know the procedure to dispose of expired medicines? | Yes | No |
| How do you dispose expired medicines? | Throw away  in dustbins  (household trash) | Flush in toilet  or sink |
|  | Return them  to pharmacies  or hospitals  for disposal | No action (keep at home) |
