# Supplementary material for: Disposal practices of unused and expired pharmaceuticals among the general public in Quetta city, Pakistan
Source: PLoS One. 2022 May 19;17(5):e0268200. doi: 10.1371/journal.pone.0268200 (PMC9119513; doi:10.1371/journal.pone.0268200)
Supplement: Attachment — Submitted filename: Response to reviewers.docx [file pone.0268200.s002.docx]

**Response to reviewers’ comments**

| **Reviewer 1** | | |
| --- | --- | --- |
| **S No** | **Reviewer’s Comment** | **Author’s Response** |
| 1. | The authors assessed the current disposal of unused and used up medicines by the general public in Quetta, Pakistan by means of a questionnaire, a typical but forgotten social health problem currently faced by the public. | We are thankful for this appreciating comment from the reviewer. |
| 2. | Suggest some feasible measures for the management of expired drugs. | Added as suggested. |
| 3. | It is suggested to classify the types of drugs before investigating the disposal. | Added as suggested. |
| **Reviewer 2** | | |
| 1. | φc was reported in the paper. Whereas original φc data was not supplied in the Tables. Could you provide them? | Φc values are added to the Table. |
| 2. | The average age of the responders was 31. Not enough aging population was included. Dose the age distribution in the questionnaire survey identified with population distribution of Pakistan? | Yes, the mean age of the respondents was 31. If we look back to Table 1, 10% of the population age was 47 years and above. We have the following justifications for the reviewer ‘query:  i) The study design was cross-sectional, and this design analyses data of variables collected at one given point in time across a sample population. We did not have a predefined population selected before the study and contacted the respondents based on convenience.  ii) Secondly, according to the data by Pakistan bureau of statistics reported in 2017, 35.3% of the Pakistani population falls into age group of 20-49 years. As our study was focused on adults, we deliberately missed out the age range of 0-17 years that comprises of 53.77% of the entire population. The aging population (above 50 years) comprises of 10.9% and that is almost equal to what is reported by our study. Therefore, based on the statistics reported in census 2017, age distribution of our study respondents is parallel to what is present in the data of census of 2017.  For details, please visit www.pbs.gov.pk/content/population-5-year-age-group-pakistan |
| 3. | More information could be collected, such as variety of the medicines unused and expired pharmaceuticals among the general public. These data should be valuable for policy setting. | We do appreciate the comment however, it is not possible at this present moment. However, we have taken the advice into consideration and have added this point in the recommendation section. |
| **Reviewer 3** | | |
| 1. | The research is well designed, well written, and logically rigorous. | We appreciate the positive comment from the reviewer. |
| 2. | However, the fit of the research topic with this journal needs to be improved. It is recommended that the author should provide necessary experimental evidence or literature support for the regulatory effects of compounds on oxidative stress and strengthen the discussion on oxidative stress. | Added as suggested. |
| 3. | It is recommended to accept the manuscript after major revision. | We appreciate the positive comment from the reviewer. |
